# Effect of Low-Dose Vitamin D Supplementation on Serum 25(OH)D in School Children and White-Collar Workers

**DOI:** 10.3390/nu9050505

**Published:** 2017-05-17

**Authors:** Ronghua Zhang, Xiamusiye Muyiduli, Danting Su, Biao Zhou, Yueqiang Fang, Shuying Jiang, Shuojia Wang, Lichun Huang, Minjia Mo, Minchao Li, Bule Shao, Yunxian Yu

**Affiliations:** 1 Zhejiang Provincial Center for Disease Control and Prevention, Hangzhou 310051, Zhejiang, China; rhzhang@cdc.zj.cn (R.Z.); dtsu@cdc.zj.cn (D.S.); bzhou@cdc.zj.cn (B.Z.); yqfang@cdc.zj.cn (Y.F.); lchhuang@cdc.zj.cn (L.H.); 2 Department of Epidemiology & Health Statistics, School of Public Health, School of Medicine, Zhejiang University, Hangzhou 310058, Zhejiang, China; xiamu@zju.edu.cn (X.M.); jiangshuying91@163.com (S.J.); wangsj2015@163.com (S.W.); 21618436@zju.edu.cn (M.M.); liminchao@zju.edu.cn (M.L.); shaobl@163.com (B.S.)

**Keywords:** serum 25(OH)D, vitamin D, supplementation, school children, white-collar workers

## Abstract

Objective: Our study aimed to investigate the nutritional vitamin D status of school children aged 9–15 years and white-collar workers in Zhejiang province, and evaluate the efficacy of low-dose-oral vitamin D supplementation in both populations. Methods: We conducted a prospective controlled trial during March 2014 to November 2015, comparing the efficacy of vitamin D supplements (400 IU/day) with non-intervention for 18 months in school children aged 9–15 years. Meanwhile, a before-after study was conducted among white-collar workers for 1 year. Serum 25(OH)D concentration was measured at baseline and after vitamin D supplementation, respectively. Results: At the baseline, 95% of school children and 84% of adult participants had vitamin D deficiency (<20 ng/mL). In school children, no difference was observed between the intervention and control groups with regard to anthropometric data. Serum 25(OH)D concentrations of the school children intervention group, school children control group and white-collar workers were 12.77 ± 3.01 ng/mL, 14.17 ± 3.59 ng/mL and 16.58 ± 3.66 ng/mL at baseline and increased to 17.34 ± 3.78 ng/mL, 18.04 ± 4.01 ng/mL and 17.75 ± 5.36 ng/mL after vitamin D supplementation, respectively. Although, after adjusting for potential confounders, the 400 IU oral vitamin D supplementation increased serum 25(OH)D concentration in school children (β = 0.81, *p* = 0.0426) as well as in white-collar workers (*p* = 0.0839), the prevalence of vitamin D deficiency was still very high among school children (79.23% in intervention group and 72.38% in control group) and white-collar workers (76.00%). Conclusions: High prevalence of vitamin D deficiency was common in these two study populations. Daily doses of 400 IU oral vitamin D supplementation was not able to adequately increase serum 25(OH)D concentrations. A suitable recommendation regarding the level of vitamin D supplementation is required for this Chinese population.

## 1. Introduction

Vitamin D belongs to a group of secosteroid molecules that are associated with bone and calcium metabolism [1]. The significant effects of vitamin D are found not only in calcium homeostasis and musculoskeletal health, but also in the integrity of the innate immune system which have been investigated extensively in recent years [2,3]. Public awareness is growing on the relative high prevalence of vitamin D insufficiency in the general population [1] which is associated with an increased risk of several diseases, including cancer [2], heart disease, hypertension [4], diabetes [5], age-related cognitive decline, Parkinson’s disease, multiple sclerosis and arthritis [3].

The major form of vitamin D in the body includes vitamin D_2_ (ergocalciferol) which is mainly obtained from dietary sources, and vitamin D_3_ (cholecalciferol) which is synthesized in the skin triggered by sun exposure, or more specifically ultraviolet B (UVB) irradiation [6]. A main source of vitamin D is the cutaneous synthesis of 7-dehydrocholesterol from sunlight exposure [7]. Serum 25-hydroxyvitamin D (25(OH)D) is the major circulating form of vitamin D, reflecting both cutaneous synthesis and dietary intake [8]. Although sufficient dietary intake of vitamin D can offset the lack of dermal synthesis, dietary sources of vitamin D are limited with fortified products [9]. Multiple interventions with vitamin D supplementation have been investigated, with variable results. A study conducted in Canadian patients with Crohn’s disease reported that a high dose of vitamin D_3_ at 10,000 IU daily significantly increased 25-hydroxy-vitamin D levels from a mean of 73.5 nmol/L to 160.8 nmol/L, while a low dose of vitamin D_3_ at 1000 IU daily did not significantly change 25-hydroxy-vitamin D levels, as values only increased from 71.3 nmol/L to 82.8 nmol/L [10]. A US study, in which female participants aged 65 years or older were given oral supplementation of 800 IU vitamin D_3_, reported that serum 25(OH) D levels rose from 13.8 to 33.1 ng/mL after 12 months and to 35.5 ng/mL after 24 months of supplementation [11]. Currently, however, there is no standard definition of the optimal serum levels of 25(OH)D. Different dosages of vitamin D intervention studies have been reported from various populations [5,12,13].

Several existing epidemiologic studies have suggested a high prevalence of vitamin D deficiency in Chinese adolescents [14,15,16]. A Chinese study conducted among children aged 6–15 years in Guangxi Province reported that vitamin D levels <30 ng/mL were detected in 85% of boys and 87% of girls. Improved understanding of vitamin D status in different groups of Chinese population will facilitate the implementation of appropriate preventative strategies [17]. The aims of our study were to investigate the nutritional status of vitamin D among school children and whiter-collar workers in Zhejiang province and to evaluate the efficacy of low-dose vitamin D intervention in school children and adults.

## 2. Materials and Methods

### 2.1. Study Design and Participants

School children participants: A prospective controlled trial was carried out in school children from March 2014 to November 2015. Participants were voluntarily recruited from primary public school situated in Jingning She nationality autonomous county, which is located in the mountainous area of southern Zhejiang Province, southern China (27°58′ N). In order to avoid contamination between groups, a class-cluster randomized trial was used to randomize the classes into an intervention class and a control class. Overall, 460 school children aged 9–15 years consented to enroll in the study.

White-collar worker participants: A before-after study was conducted in white-collar workers from July 2013 to July 2014. Sixty-one white-collar workers aged 24–65 years were involved, who mainly worked in offices and performed tasks requiring mental effort, living in Hangzhou, the capital city of Zhejiang province.

All participants were ethnic Han Chinese. Exclusion criteria included the following conditions: already taking vitamin D supplements or related fortified foods; had an infectious disease in the past three months; suffered from any major diseases (Cushing syndrome, acromegaly, severe anemia, primary hypothyroidism or hyperthyroidism, epilepsy, malignant tumors); had acute and chronic gastritis, gastric ulcer or other digestive system diseases; had rheumatic or congenital heart disease; had a resting heart rate <45/min or >100/min; liver function was significantly abnormal oralanine aminotransferase (ALT) was three times higher than the normal upper limit; had evidence of renal dysfunction or serum creatinine two times higher than the normal upper limit; needed long-term use of statins or glucocorticoid drugs; self-reported to be HIV-positive; or reported any other obvious diseases. Written informed consent was obtained from each participant or legal guardian for the school children, and the study protocol was approved by the Medical Ethics Review Committee of Zhejiang Provincial Center for Disease Control and Prevention (2013-002).

### 2.2. Interventions and Procedures

All eligible school children participants were asked to complete a questionnaire at baseline, which include demographic characteristics, disease history, daily lifestyle and family information. After over-night fasting, 5 mL peripheral venous blood were collected into a vacuum-evacuated tube containing ethylenediaminetetraacetic acid (EDTA) anticoagulation from each participant and were subsequently centrifuged at 1000 rpm/min for 10 min. Supernatants were stored at −80 °C, until they were used to measure baseline vitamin D concentrations. Participants in the intervention group were provided with vitamin D supplements and instructed to orally take one tablet daily during the study period (each tablet contianed 400 IU vitamin D_3_. Vitamin D supplementation tablets were uniformly from Sinopharm Xingsha Pharmaceuticals Co. Ltd., Xiamen, China). Participants in the control group were not provided with any intervention. For ensuring the compliance, we conducted one telephone call weekly to school children’s parents to remind participants to take daily supplementation. Additionally, while the dispensing of vitamin D tablets was conducted monthly, the investigators also reminded participants to take the vitamin D tablet in a timely fashion. After the 18-month intervention, each child completed a standardized questionnaire including demographics, lifestyle factors, physical activity, dietary intake, and fortified food supplementations. Additionally, 5 mL fasting peripheral venous blood was sampled.

At baseline, white-collar worker participants filled in a well-structured questionnaire including sociodemographic characteristics, lifestyle factors, and dietary information. Fasting peripheral venous blood samples were collected in the same way as for the school children participants. All of the 61 white-collar workers received vitamin D supplements, and instructed to orally take one tablet (each tablet contained 400 IU vitamin D_3_) daily for one year. We conducted telephone calls weekly to remind participants to maintain their daily supplementation. While the dispensing of vitamin D tablets was conducted monthly, investigators also regulated the intake of vitamin D tablets. Both before and after the intervention, fasting peripheral venous blood samples were collected. The sample collection, transportation and preservation were carried out in strict accordance with regulations. Routine procedures were operated by professionals, and investigation instructors were in charge of quality control, thus ensured the accuracy and reliability of the test results.

### 2.3. Measurements

Anthropometric measurements were performed by trained healthcare workers; body mass index (BMI) was calculated as weight in kilograms divided by the square of the height in meters (kg/m^2^). Dietary intake assessments were conducted by standardized food portions after the 18 months’ follow-up in school children and at baseline in white-collar workers, respectively. Food variety score was measured as the total number of food items consumed over a year [18]. Serum 25(OH)D (comprised of 25(OH)D_2_ and 25(OH)D_3_) concentrations were quantitatively determined by using a commercially available enzyme immunoassay kit (OCTEIA 25-(OH)D Kit, Immuno Diagnostic Systems, Boldon, UK) at the laboratory of Zhejiang Provincial Center for Disease Control and Prevention. Precision testing showed within-run coefficient of variations (CVs) of ≤7%, within-laboratory CVs of <9.5%, and between-laboratory precision CVs of ≤10.1% [19]. Samples randomized across plates. Quality control materials were analyzed every day to test if the measuring value was within analytical measuring range. Standard control was used for the adjustment of measuring curve when the batch of reagent was changed.

### 2.4. Statistical Analysis

The continuous and categorical variables were presented as means (standard deviation) and frequencies (percentage), respectively. A comparison of the demographic characteristics, anthropometry information and clinical examination between the intervention and control groups were conducted in school children participants using two-sample *t*-tests and chi-square tests, respectively. Within group changes of the serum 25(OH)D concentrations in white-collar workers was tested using paired *t*-tests. Linear regression analyses were applied to quantify the associations between vitamin D supplementation and serum 25(OH)D concentrations. In the multivariable regression models, gender, age, BMI, physical activity, outdoor activity time and food variety score were taken as potential confounders. All data from the questionnaires were entered in Epidata 3.0 (Epidata Association, Odense, Denmark). Data analyses were performed using Statistical Analysis System software version 9.2 (SAS Institute, Cary, NC, USA). The statistically significant level was *p* < 0.05.

## 3. Results

### 3.1. Participant Characteristics

In this study, 460 school children were enrolled at the baseline, and 38 (8.3%) of them excluded from final analyses due to incomplete data acquisition at the end of the follow-up. Overall, 422 school children aged 9–15 years (208 boys, 214 girls) were included. Demographic characteristics are shown in Table 1; distributions of gender, age, BMI, blood pressure and hemoglobin concentration did not show significant difference between the intervention group and the control group. A total of 61 white-collar workers were enrolled at the baseline, and 50 of them (16 men, 34 women) who had complete data were included in the final analyses. Table 2 summarizes the general characteristics of the white-collar workers. Adult participants were aged 24–65 years (41.69 ± 12.69). BMI was 22.68 ± 2.76 kg/m^2^, systolic blood pressure (SBP) and diastolic blood pressure (DBP) were 113.04 ± 11.81 mmHg and 71.68 ± 8.97 mmHg, respectively.

### 3.2. Serum 25(OH)D Levels at Baseline and Follow-Up

School children’s serum 25(OH)D concentrations at the baseline and after the 18-month follow-up are presented in Table 3. The mean baseline serum 25(OH)D concentration was 13.56 ± 3.42 ng/mL among school children. At the baseline, a total of 402 (95%) school children suffered from vitamin D deficiency, which including 180 school children in the intervention group (98.36% of 183) and 222 in the control group (92.89% of 239). The control group had a significantly higher mean serum 25(OH)D concentration compared with intervention group at the baseline (*p* < 0.0001). After the 18 months’ vitamin D supplementation, serum 25(OH)D concentrations increased from 12.77 ± 3.01 to 17.34 ± 3.78 ng/mL in the intervention group and from 14.17 ± 3.59 to 18.04 ± 4.0 ng/mL in the control group. Serum 25(OH)D in the two groups increased about 4.57 ± 3.60 ng/mL and 3.87 ± 3.81 ng/mL, respectively. Paired *t*-test analysis found that serum 25(OH)D concentrations of before and after the intervention in each group increased in both groups (*p* < 0.0001). However, there was a marginally significant difference in the increment of serum 25(OH)D concentrations between the intervention group and the control group (*p* = 0.0544). At the end of the study, 145 school children in the intervention group (79.23% of 183) and 173 in the control group (72.38% of 239) still suffered from vitamin D deficiency (<20 ng/mL).

In white-collar workers, the mean baseline serum 25(OH)D concentration was 16.58 ± 3.66 ng/mL, 38 (84.00%) of adults had vitamin D deficiency (<20 ng/mL). After vitamin D intervention, serum 25(OH)D concentrations increased from 16.58 ± 3.66 to 17.75 ± 5.36 ng/mL. Of the participants, 38 (76.00%) adults still had vitamin D deficiency (<20 ng/mL). A total increment of 1.16 ± 4.68 ng/mL was observed, which showed no significant difference between before and after the intervention (*p* = 0.0839). Subgroup analyses showed that baseline serum 25(OH)D concentrations in males were significantly higher than that of females. Twenty-four older participants (≥40 years) had significant changes of serum 25(OH)D concentrations before and after the vitamin D intervention (*p* = 0.0209) (Table 4).

### 3.3. Relationship between Vitamin D Supplementation and Serum 25(OH)D Concentrations in School Children

The association of vitamin D supplementation with serum 25(OH)D concentrations in school children are shown in Table 5. In different multivariable regression analyses models, the baseline serum 25(OH)D concentrations of the intervention group were lower than that of the control group (β = −1.52, *p* < 0.0001), and difference persisted after adjustment for age, gender, BMI, physical activity, outdoor activity time and food variety score (β = −1.49, *p* < 0.0001). After vitamin D supplementation, the crude model showed no significant difference in serum 25(OH)D concentrations between the intervention group and the control group (β = −0.69, *p* = 0.0718). After adjustment for age, gender and BMI in model 2, the difference became significant (β = −0.76, *p* = 0.0496), but in model 3 the difference turned out to be insignificant when further adjusted by physical activity, outdoor activity time, food variety score and baseline serum 25(OH)D concentrations (β = 0.04, *p* = 0.9134). The adjusted models 2 and 3 both showed a positive association between vitamin D supplementation and the increment of serum 25(OH)D concentrations (β = 0.76, *p* = 0.0444; β = 0.81, *p* = 0.0426, respectively).

## 4. Discussion

In this study, vitamin D status in prospective interventional cohorts of children and adults were assessed. Our results indicated that both the school children and white-collar workers had low mean serum 25(OH)D concentrations at baseline (13.56 ± 3.42 ng/mL and 16.58 ± 3.66 ng/mL). After vitamin D supplementation, significant difference was detected in changes of serum 25(OH)D concentrations in school children between the intervention vs. control groups (*p* = 0.0426). This was also confirmed in white-collar workers, as a marginally significant difference was found between baseline vs. follow-up serum 25(OH)D concentrations (*p* = 0.0839). In spite of the small extent of the increments of serum 25(OH)D concentrations, we did not find that low-dose (400 IU/day) vitamin D supplementation could significantly improve vitamin D deficiency in the children and adult participants.

The serum 25(OH)D levels in the present study were much lower than other observed populations reported in previous domestic investigations [15,20]. A survey conducted in Hangzhou (30° N), revealed that school children aged 6–11 years had a mean serum 25(OH)D concentration of 56 nmol/L (22.4 ng/mL), and adolescents aged 12–16 years old had a mean concentration of 52 nmol/L (20.8 ng/mL) [21]. Another study in Huzhou (30.86° N) showed that children suffered from low vitamin D status, and mentioned that from 1 year to 17 years of age, it decreased continuously and significantly with age [22]. Other previous studies, however, mentioned lower 25(OH)D levels from different locations around the world [21,22,23,24,25,26].

As a worldwide prevalence of vitamin D deficiency has been reported in recent decades, vitamin D status varies across different populations and regions [27,28,29,30]. During periods of rapid skeletal growth in childhood, school children are in great demand of nutrients including vitamin D. Observational studies have suggested that a large part of children have suboptimal levels of 25(OH)D [31,32,33]. There is still no consensus on the optimal level of serum 25(OH)D concentrations for healthy children or adults. The US Endocrine Society defined vitamin D deficiency as a 25(OH)D level below 20 ng/mL (50 nmol/L), and vitamin D insufficiency as a 25(OH)D level of 21–29 ng/mL (52.5–72.5 nmol/L) in 2011 [34]. The Institute of Medicine (IOM) released different reference values; 97.5% of the general population are assured bone health when serum 25OHD levels are ≥20 ng/mL (50 nmol/L), while serum 25(OH)D concentrations <16 ng/mL (40 nmol/L) reflect a level that is sufficient to ensure bone health for approximately 50% of the general population [35,36,37]. The differences may have resulted from different laboratory assays to measure serum 25(OH)D levels, hence, the quality controls varied across different studies. In the current study, 25(OH)D levels were measured in March (baseline) and then in November (follow-up). The serum 25(OH)D level varied remarkably with the change in seasons, which was highest in summer among the Chinese population [15,22]. While baseline and final blood were sampled in different seasons, the use of the intervention and control groups allowed for comparisons between groups under the assumption that exposure to vitamin D across seasons was similar.

White-collar workers were examples of the adult population who mainly worked indoor, lacking sufficient sun exposure and engaging less leisure-time activity. They are also in great demand of nutrients like vitamin D. Vitamin D deficiency may result in rickets among children [38] and osteomalacia among adults [39]. All of our study samples were from an apparently healthy population, although the mean serum 25(OH)D levels of the sample population were lower than other reports, and any vitamin D-related subclinical signs were not found during study period. In white-collar workers aged >40 years, the mean serum 25(OH)D concentration increased significantly after the intervention, but 76% of adults still had vitamin D deficiency (<20 ng/mL). Hence, low dose vitamin D supplementation did not significantly improve vitamin D deficiency in the adult participants.

In the multivariable analyses, the unadjusted model showed no significant correlation in changes of 25(OH)D concentrations between the baseline and follow-up with vitamin D supplementation among school children. However, because the baseline 25(OH)D concentrations were not comparable, baseline 25(OH)D concentrations were taken as covariates in the multivariable model, and the 25(OH)D concentrations after the intervention in the intervention group were significantly higher than those of the control group. However, the deficiency of vitamin D was also not obviously improved in the intervention group (79.23%). A meta-analysis showed that there was no significant difference in 25(OH)D levels between the lower dose (<800 IU/day) supplementation versus the placebo among adolescents; and the author suggested that an intermediate vitamin D dose of 1000–2000 IU daily may be necessary to allow for the majority of the population to reach a desirable 25(OH)D level of 20 ng/mL [40]. A study which assessed high dose vitamin D supplementation safety reported that a 2000 IU/day intervention for 1 year resulted in desirable vitamin D levels in adolescents [12].

Dietary intake of vitamin D is known to have a smaller contribution to vitamin D status than cutaneous production in response to ultraviolet light exposure. The Pediatric Branch of the Chinese Medical Association recommends a daily vitamin D supply of 400 IU for children aged 0~6 years, and 100 IU for children over 7 years of age [41]. The Scientific Advisory Committee on Nutrition in the UK advocated a reference nutrient intake of 400 IU vitamin D daily for the general population aged over 4 years, and an upper limit of 4000 IU/day [13]. The US IOM Committee’s suggested the AI (adequate intake) in infancy is estimated to be 400 IU/day. The RDA (dietary reference intake) is 600 IU/day for people >1 year old, except men and women aged 71 and older (for whom the RDA is 800 IU/day) [36]. Inappropriate vitamin D supplementation escalates healthcare costs and may cause adverse effects. Therefore, it is necessary to certify a moderate dose of vitamin D supplementation fit for the Chinese population.

A the school children participants of the present study were from the mountainous area of Zhejiang Province, our findings may not generalize to the population in other areas, and extrapolating the conclusions needs careful consideration. Additionally, a confounding bias may exist in this study. Class-cluster randomization was applied in school children. However, as individual randomization was not applied, it might result in a confounding bias between the intervention and control groups. The adult study lacked a control group, so there remains the possibility that variation in environmental and dietary factors produced the effect on the vitamin D level. So, these results from the before-after study design might not be robust. Although we conducted telephone follow-up calls weekly to remind participants to maintaining their daily supplementation, compliance in some participants might not have been conducted very well. Also, the small sample size might produce unstable results in adults.

## 5. Conclusions

Our study demonstrated a high prevalence of vitamin D deficiency among school children and white-collar workers. This study indicated that a daily dose of 400 IU of oral vitamin D supplementation cannot efficaciously elevate serum 25(OH)D concentrations, and that prevention of vitamin D deficiency may require a higher dose of vitamin D supplementation or more sun-exposure among school-aged children in mountainous area and indoor workers.

## Figures and Tables

**Table 1:** Characteristics of the intervention and control groups in school children.

Variables	Total Participants (*N* = 422)	Intervention Group (*N* = 183)	Control Group (*N* = 239)	*p* Value
Gender, *N* (%)				
Male	208 (49.29)	88 (48.09)	120 (50.21)	0.6657
Female	214 (50.71)	95 (51.91)	119 (49.79)	
Age (years)	11.83 ± 1.68	11.74 ± 1.69	11.90 ± 1.67	0.3231
BMI (kg/m^2^)	18.94 ± 3.07	18.87 ± 3.06	18.99 ± 3.08	0.2220
SBP (mmHg)	109.05 ± 11.09	108.40 ± 11.29	109.60 ± 10.94	0.2752
DBP (mmHg)	65.87 ± 7.55	65.22 ± 7.57	66.38 ± 7.51	0.1172
Hemoglobin (g/L)	130.64 ± 9.82	129.70 ± 9.41	131.4 ± 10.08	0.0775
Follow-up BMI (kg/m^2^)	20.02 ± 3.54	20.14 ± 3.47	19.93 ± 3.59	0.0718

**Table 2:** Characteristics of white-collar workers.

Variables	Participants (*N* = 50)
Gender, *N* (%)	
Male	16 (32.00)
Female	34 (68.00)
Age (years)	41.69 ± 12.69
BMI (kg/m^2^)	22.68 ± 2.76
SBP (mmHg)	113.04 ± 11.81
DBP (mmHg)	71.68 ± 8.97

**Table 3:** Comparison of changes in serum 25(OH)D before and after the intervention among school-children (ng/mL).

	Total Participants (*N* = 422)	Intervention Group (*N* = 183)	Control Group (*N* = 239)	*p* Value ^a^
Baseline	13.56 ± 3.42	12.77 ± 3.01	14.17 ± 3.59	<0.0001
Follow-up	17.73 ± 3.92	17.34 ± 3.78	18.04 ± 4.01	0.0718
Difference	4.17 ± 3.73	4.57 ± 3.60	3.87 ± 3.81	0.0544
*p* value ^b^	<0.0001	<0.0001	<0.0001	

^a^ Intervention vs. control group; ^b^ Baseline vs. follow-up.

**Table 4:** Comparison of changes in serum 25(OH)D before and after the intervention among the white-collar worker study population (ng/mL).

Variables	*N*	Baseline	Follow-Up	Difference	Baseline vs. Follow-Up *p* Value
Total participants	50	16.58 ± 3.66	17.75 ± 5.36	1.16 ± 4.68	0.0839
**Gender**					
Male	16	18.57 ± 4.36	20.22 ± 6.80	1.65 ± 6.07	0.2918
Female	34	15.64 ± 2.91	16.58 ± 4.16	0.93 ± 3.94	0.1756
*p* value *		0.0073	0.0621	0.6677	
**BMI (kg/m^2^)**					
≤24	34	16.57 ± 3.79	17.82 ± 5.84	1.24 ± 5.04	0.1610
>24	16	16.59 ± 3.51	17.60 ± 4.34	1.01 ± 3.91	0.3190
*p* value *		0.9867	0.8967	0.8713	
**Age (years)**					
≤40	26	16.49 ± 3.75	16.76 ± 5.12	0.27 ± 4.97	0.7812
>40	24	16.68 ± 3.64	18.82 ± 5.52	2.13 ± 4.21	0.0209
*p* value *		0.8514	0.1780	0.1621	

* Within group *p* value.

**Table 5:** Adjusted association of vitamin D intervention with 25(OH)D concentrations among school children (*N* = 422).

Variables	Model 1		Model 2		Model 3	
	β(se)	*p* Value	β(se)	*p* Value	β(se)	*p* Value
Baseline						
Control group	REF		REF		REF	
Intervention group	−1.39 (0.33)	<0.0001	−1.52 (0.33)	<0.0001	−1.49 (0.34)	<0.0001
Follow-up						
Control group	REF		REF		REF	
Intervention group	−0.69 (0.38)	0.0718	−0.76 (0.38)	0.0496	0.04 (0.37)	0.9134
Increment						
Control group	REF		REF		REF	
Intervention group	0.70 (0.36)	0.0544	0.76 (0.38)	0.0444	0.81 (0.39)	0.0426

Model 1: Crude; Model 2: Adjustment for age, gender and BMI; Model 3: Adjustment for age, gender, BMI, physical activity, outdoor activity time and food variety score, (follow-up model also adjusted baseline 25(OH)D).
